# Understanding the association of disability with multimorbidity, and healthcare utilization in India’s older adult population: insights from cross-sectional evidence of SAGE-2

**DOI:** 10.3389/fpubh.2024.1435315

**Published:** 2024-10-23

**Authors:** Ritik Agrawal, Abhinav Sinha, Jogesh Murmu, Srikanta Kanungo, Sanghamitra Pati

**Affiliations:** ^1^ICMR-Regional Medical Research Centre, Bhubaneswar, India; ^2^South Asian Institute of Health Promotion, Bhubaneswar, India

**Keywords:** disability, multimorbidity, WHODAS 2.0, SAGE, healthcare utilization, India

## Abstract

**Background:**

Like other low-and middle-income countries, India is undergoing a demographic and epidemiologic shift that has led to a significant rise in the burden of non-communicable diseases (NCDs). Evidence suggests that chronic illnesses and disability are linked but limited studies have explored the association between disability and multimorbidity (simultaneous presence of two or more chronic conditions). Since the magnitude of multimorbidity is becoming a norm, it is prudent to understand the association between these two. We aimed to estimate the association between disability and multimorbidity and assess their healthcare utilization among older adults in India using a nationally representative data.

**Methods:**

We employed data from the second round of World Health Organization’s Study on Global AGEing and adult health (SAGE) conducted in 2015. SAGE is a nation-wide survey conducted among a representative sample of older adults aged ≥50 years, a total of 7,118 participants aged ≥50 years were included in the analysis. The main outcome of interest was disability for which we used WHODAS 2.0 scoring scale which ranges from 0 to 100. Descriptive statistics such as frequency and proportion were used to report the characteristics of study population, and prevalence. We performed the univariable followed by multiple ordinal logistic regression to assess the association between disability and multimorbidity, reported as adjusted odds ratio (AOR) with 95% confidence interval (CI) and *p*-value. Healthcare utilization was presented as frequency and proportion.

**Results:**

The overall prevalence of disability was found to be 89.0% (95% CI: 88.3–89.8) while that of multimorbidity was 39.7% (95% CI: 35.6–37.8). Most of the participants had moderate followed by mild disability. Hypertension (32.7%) was found to be the most prevalent chronic condition followed by cataract (21.3%). The chances of having disability among multimorbid individuals was AOR: 1.40 (95% CI: 1.13–1.75). Participants having disability and multimorbidity mostly visited private sector followed by public healthcare facilities.

**Conclusion:**

We observed that nine out of every 10 individuals had some kind of disability in India. We observed multimorbidity to be associated with disability that signifies the need for including disabilities as a part of NCD program as these factors could be bi-directional. Longitudinal studies for disability will be helpful to better understand and address the growing needs of these individuals.

## Introduction

Declining trends of fertility, increased life expectancy, increased survival at old ages and migration to some extents are some of the driving factors responsible for aging of population, globally (1). According to UN Population forecasts, the proportion of Indians aged 60 and more would rise from 8% currently to 19% by 2050 (2). Multimorbidity encompasses two or more chronic ailments without considering any index condition. Multimorbidity becomes increasingly prevalent with age and is more prevalent among the older adults (estimated between 20 and 23% for adults aged 18 years to those above 45 years of age) (3). Multimorbidity presents a particular challenge to healthcare professionals in providing care to older (4). Previous research has revealed that multimorbidity is related with adverse health consequences, including mortalities, disabilities, and poor quality of life (5). A population-based study in Telangana revealed that every third individual had at least one non-communicable disease (NCD) and every fifth older adult had at least one disability (6).

The World Health Organization (WHO) provides a definition of disability, describing it as an impairment, limitation, or restriction in activity primarily resulting from health conditions and environmental factors (7). There is a positive correlation between functional and physical disability and the presence of chronic and co-existing illnesses (8, 9). In low- and middle-income countries (LMICs), NCDs such as cardiovascular and musculoskeletal disorders contribute to approximately 66.5% of disability-adjusted life years (DALYs) (10, 11). Age-related conditions were responsible for almost 51% of the years of life lost (YLL) and years lived with disability (YLD) in 2017 (12, 13). Despite the increasing predominance of multimorbidity among the older adults, modern medical research and practice predominantly focuses on the single disease model and give little consideration to coexistence of many diseases. However, as multimorbidity research has expanded into disability it was revealed that the negative influence of multimorbidity on disability increases with increase in the number of chronic illnesses (14, 15).

The majority of elder impairments are preventable or treatable. With timely and proper care, the older adult can become more functional and could improve their quality of life. Disability is a big barrier to older adults receiving care (16). Evidence suggests that chronic illnesses and disability are linked but limited studies have explored the association between disability and multimorbidity. Since the magnitude of multimorbidity is becoming a norm, it is prudent to understand the association between these two. Another aspect of having multiple long-term conditions is increased visit to different specialists, followed by separate investigations and buying multiple medicines that brings financial burden upon families. Besides financial concerns, side-effects of polypharmacy include adherence, drug reaction and over-prescription or misuse (17).

In a LMICs such as India the migration of young care givers to urban cities compels the older adults for self-care. As individuals’ health deteriorates due to a rising number of chronic diseases, various physical and psychological issues arise, affecting their overall wellbeing (18). It underlines the importance of exploring the prevalence of disabilities, and well as its association with multimorbidity particularly among older adults. Additionally, it is critical to understand the interaction among multimorbidity and disability in order to formulate long-term healthcare policies and programs, minimizing functional disability, and to improve the health-related life quality (HRQoL) among this group.

Presently, primary healthcare in India is administered through a combination of public and private systems (19). The government heavily finances the public healthcare sector, and patients contribute a nominal fee for pharmaceuticals, diagnostics, or treatments (20). Conversely, private healthcare operates on a fee-for-service basis, requiring patients to cover costs directly or through employer or insurance funding. Despite the elevated expenses associated with private healthcare, recent national sample surveys highlight that 70% of patients in India seek private healthcare either in conjunction with or alongside public health services (21). The elevated costs involved may result in increased financial burdens; hence, there is a necessity to generate relevant evidence on healthcare utilization among individuals experiencing both disability and multimorbidity. Therefore, we aimed to estimate the association between disability with multimorbidity and assess their healthcare utilization among older adults in India using a nationally representative data.

## Methods

### Overview of data

The data employed in this study was sourced from the 2015 s round of the WHO Study on Global Ageing and Adult Health (SAGE). SAGE is a nationwide survey that includes a representative sample of older adults aged ≥50 years and a smaller cohort of adults aged 18–49 years. This comprehensive longitudinal study gathers information on the health and well-being of adults and captures the aging dynamics in six countries: India, China, Russia, Ghana, Mexico, and South Africa. In the present study we used data for India only. Data collection in India was carried out in six states, namely Assam, Maharashtra, Karnataka, Rajasthan, Uttar Pradesh and West Bengal. The study employed a multistage stratified cluster random sampling design to ensure representative observations. SAGE employed community-based, in-person interviews, employing standardized survey instruments for data collection. Prior to data collection, the staff underwent comprehensive training to ensure consistent and accurate data assimilation. For a comprehensive understanding of the survey methods employed during SAGE, the India National report of SAGE, wave-2 provides a detailed description (22).

### Study participants and sample size

The SAGE survey, wave-2, in India covered a total of 11,818 individuals. To ensure consistency with our research objectives, we excluded 1,998 participants aged below 50 years. It’s important to highlight that SAGE’s focus was on providing a representative sample of respondents aged ≥50 years, while individuals below 50 years constitute a smaller subgroup that was not considered in our analysis. Additionally, 2,702 individuals having missing values were also dropped. As a result, the analysis was conducted with a total of 7,118 participants aged ≥50 years.

A two-stage sampling technique was used for rural regions, and main sample units (villages) were selected using the probability proportional to size method, using the population of the village as the measure of size from the 2001 Census. Systematic sampling was used to choose the secondary sampling units (households), and Kish tables were used to choose the tertiary sampling units (individuals). A three-stage sample procedure was utilized for urban areas, with the primary sampling units (city wards) chosen using the probability proportional to size technique. By randomly picking two from each PSU, secondary sample units (also known as census enumeration blocks) were selected. Systematic sampling was used to choose the secondary sampling units (households), and the primary sample unit (individuals) was selected in the same way as in rural regions. Thus, a total of 379 EAs were chosen as the principal sample units (PSUs) (22).

### Outcome variable

Our main outcome of interest centered around disability, and we employed the World Health Organization’s Disability Assessment Schedule (WHODAS-2.0) scoring scale, with a ranging from 0 to 100. Disability was assessed using a comprehensive set of 12 distinct questions that align with the WHODAS 2.0 framework. This standardized instrument is designed to capture limitations experienced in activities and daily social participation over last month. The WHODAS 2.0 encompasses six domains of functioning, namely (1) comprehension and communication, (2) self-care, (3) mobility, (4) interpersonal relations, (5) domestic and work roles (life activities), and (6) community as well as civic roles (participation). The scores for each domain in the WHODAS 2.0 were determined using a five-point Likert scale, with a rating of “none” equating to a score of 1, “mild” equating to 2, “moderate” equating to 3, “severe” equating to 4, and “extreme” equating to 5 as evident from previous literature (23, 24). These questions were taken and WHODAS 2.0 scale was made with scoring of 0 to 100 (24). The questions include cognition, interpersonal relations and functional assessment. A normalized score of ≥25 was used to define clinically significant disability. Additionally, we divided the disability into five categories with a score (0/4 into “No disability”) (5/24 into “Mild”) (25/49 into “Moderate”) (50/95 into “Severe”) and (96/100 into “Extreme”) (24). The detailed description of the variables is given in Table 1.

**Table 1:** Characteristics of study population (*N* = 7,118).

Variables		Unweighted *n* (%)
Age (years) (*n* = 7,118)	50–59	2,904 (40.8%)
	60–69	2,585 (36.3%)
	70–79	1,285 (18.0%)
	≥80	344 (4.8%)
	Mean ± SD	62.7 ± 8.9
Sex (*n* = 7,118)	Male	3,337 (46.9%)
	Female	3,781 (53.1%)
Residence (*n* = 7,118)	Rural	5,091 (71.5%)
	Urban	2,027 (28.5%)
Education (*n* = 7,118)	No formal education	3,504 (49.2%)
	Been to school	3,614 (50.8%)
Occupation (*n* = 7,109)	Never worked	1,887 (26.5%)
	Worked	5,222 (73.5%)
Partner status (*n* = 7,118)	Have partner	5,308 (74.6%)
	No partner	1,810 (25.4%)
Wealth index (*n* = 7,118)	Most deprived	1,371 (19.3%)
	2	1,304 (18.3%)
	3	1,318 (18.5%)
	4	1,468 (20.6%)
	Most affluent	1,657 (23.3%)
Multimorbidity (*n* = 7,118)	Present	2,613 (36.7%)
	Absent	4,505 (63.3%)
Self-rated health (*n* = 7,108)	Very good	305 (4.3%)
	Good	2,189 (30.8%)
	Moderate	3,396 (47.8%)
	Bad	1,127 (15.9%)
	Very bad	92 (1.3%)

### Independent variables/covariates

The age of the respondents was grouped into four distinct categories: 50–59 years, 60–69 years, 70–79 years, and ≥80 years. Their gender was recorded as either male or female. Regarding their place of residence, participants either belong to the urban or rural areas. The educational status of the respondents was divided into two groups: ‘no formal education’ and ‘been to school,’ based on the question “have you ever been to school?.” Respondent’s occupation was determined by the question “have you ever in your life done any type of work (not including housework)?” with the possible responses being ‘yes’ or ‘no.’ Those who responded ‘yes’ were categorized as ‘worked’, while those who answered ‘no’ were grouped as ‘never worked’. Partner status was determined based on the question “what is your current marital status?” Respondents who were currently married or cohabiting were grouped as ‘have partner’, while those who had never married, were separated/divorced, or widowed were classified as ‘no partner.’ The wealth index was divided into quintiles, representing the most deprived, 2nd, 3rd, 4th, and the most affluent classes.

Multimorbidity (two or more chronic conditions in an individual) was generated by simple sum of all chronic conditions present in an individual, i.e., chronic disease score. We included a total of 10 chronic conditions with nine self-reported chronic conditions: arthritis, stroke, diabetes, chronic lung disease, asthma, depression, hypertension, cataract, and edentulism. Additionally, obesity was calculated by considering weigh in kg divided by height in m^2^ using cut off for WHO’s body mass index (BMI) for South Asian adults (25 kg/m^2^) (25). The measurement of self-rated health was based on responses to the question “In general, how would you rate your health today?” Participants provided their ratings, which were then categorized as very good, good, moderate, bad, and very bad.

To assess healthcare utilization, the question “What was the last (most recent) health care facility you visited in the last 12 months?” was employed. Responses were categorized as follows: private doctor’s office, private clinic/health care facility, and private hospital were grouped as ‘private’; public clinic or health care facility and public hospital were categorized as ‘public’; charity/church-run clinic and charity/church-run hospital were classified as ‘charitable clinics,’ and ‘others’ included home visits. A comprehensive breakdown of the variables is available in Supplementary Table S1.

### Statistical analysis

Data was analyzed using STATA v16.0 (Stata Corp, Texas). We presented continuous data such as age in mean and standard deviation. Descriptive statistics such as frequency and proportion were used to report the characteristics of study population, and prevalence. All analyses were performed using survey weights to account for the complexities of the survey design. Weighted proportions were reported along with their corresponding 95% confidence intervals (CI) to provide a measure of uncertainty. The disability variable categorized into five levels, was analyzed using ordinal logistic regression to appropriately account for the ordered nature of the categories. Univariate ordinal logistic regression was first employed to examine the relationship between disability and various socio-demographic factors, with results expressed as odds ratios (OR) and 95% CI. To further explore these associations, multiple ordinal logistic regression model was developed, adjusting for potential confounding variables. The findings are presented as adjusted odds ratios (AOR), along with 95% CIs and *p*-values. The overall healthcare utilization of multimorbid individuals as well as individuals with multimorbidity as well as disability was shown in form of frequency and proportions.

### Ethical consideration

The analysis was based on anonymous secondary data procured through legitimate means, effectively addressing privacy concerns. The SAGE was granted approval from the Ethics Review Committee by WHO, and in the Indian context, ethical approval was secured from the International Institute of Population Sciences, Mumbai, which served as the collaborating institution for conducting this study in India.

## Results

### Description of the socio-demographic characteristics

Among our study population almost four of 10 individuals belonged to the age group of 50–59 years (40.8%). The mean age of respondents was 62.7 ± 8.9 years ranging from 50 to 101 years. More than half of the respondents were females (53.1%) and had been to school (50.8%). Almost three fourth of the respondents resided in rural areas (71.5%) and were working (73.5%). Multimorbidity was prevalent in 36.7% of the individuals and almost 47.8% of them rated their health as moderate (Table 1).

### Prevalence pattern of different disabilities

**Figure 1:**
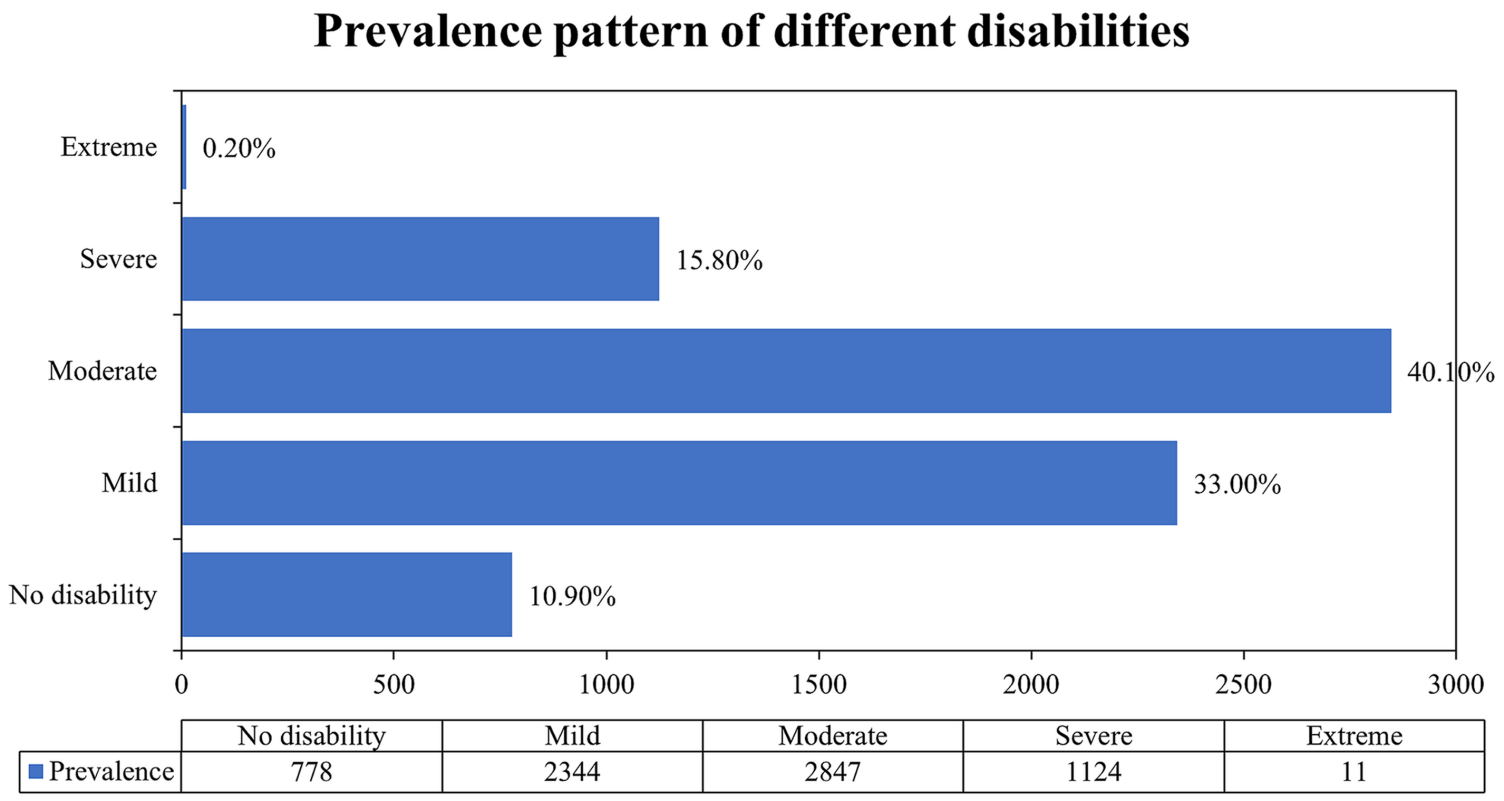
Prevalence pattern of different disabilities as defined by the World Health Organization Disability Assessment Schedule (WHODAS 2.0). Disability was also further categorized into five ordinal categories based on normalized scores [none (0–4), mild (5–24), moderate (25–49), severe (50–95), and complete (96–100)] (24).

The overall prevalence of disability was found to be around 89.0% (95% CI: 88.3–89.8). Almost half of the participants had moderate disability 40.10% (95% CI: 38.9–41.2) followed by mild 33.0% (95% CI: 31.9–34.1) and a very small proportion of them had extreme disability 0.20% (95% CI: 0.7–0.27) (Figure 1).

The analysis of various functional limitations, as measured by the mean scores across various disability domains, reveals significant variability in the impact of different activities on older adults. Among the highest reported limitations were learning new tasks (mean score 1.4), walking (mean score 1.38), and standing for long periods (mean score 1.33), indicating that physical tasks and cognitive challenges represent the greatest barriers to daily functioning. Emotional and cognitive domains, such as being emotionally affected and concentrating, both recorded a mean score of 1.1, further highlighting the multidimensional nature of disability that spans both physical and psychological aspects.

**Figure 2:**
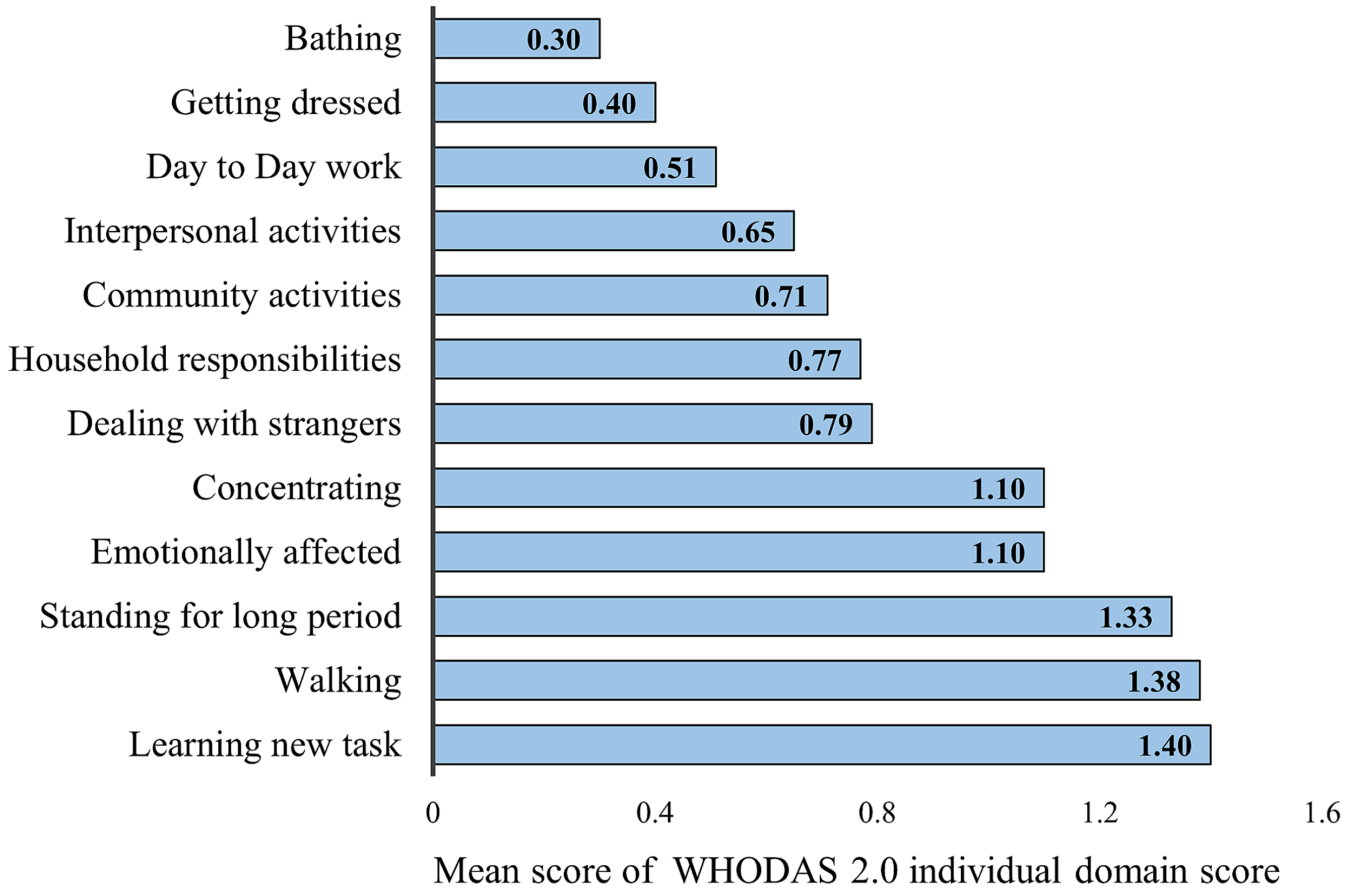
Mean score of all the individual domains of World Health Organization Disability Assessment Schedule (WHODAS 2.0) disability domain scores. Each domain of the (WHODAS 2.0) was scored on a five-point Likert scale (none = 1; mild = 2; moderate = 3; severe = 4; and extreme = 5), as previously described (24).

In contrast, activities related to basic self-care, such as bathing (mean score 0.3) and getting dressed (mean score 0.4), had the lowest mean scores, suggesting that, while physical mobility may be compromised, a relatively smaller proportion of the population experiences severe limitations in performing personal care tasks. Intermediate difficulties were reported in areas such as dealing with strangers (mean score 0.79), household responsibilities (mean score 0.77), and community activities (mean score 0.71), pointing to challenges in maintaining social engagement and managing everyday responsibilities. The detailed mean score of all domains of disability is given in Figure 2.

### Profile of chronic conditions

Among the multimorbid individuals, it was evident that disability was present in almost 96.2% (95% CI: 95.4–97.0) participants. Hypertension was the most prevalent chronic condition 32.7% (95% CI: 31.5–33.8) followed by cataract 21.3% (95%CI: 20.3–22.2) whereas depression 1.8% (95% CI: 1.5–2.2) was found to be the least prevalent among the respondents. Among all the chronic conditions, disability was most prevalent among the respondents diagnosed with stroke 98.9% (95% CI: 96.4–99.9) followed by asthma 98.8% (95% CI: 97.0–99.7). In contrast to this. Disability was found to be least prevalent among the obese individuals 91.9% (95% CI: 90.3–93.4) (Table 2).

**Table 2:** Profile of chronic conditions.

Chronic Condition	Prevalence *n*, % (95% CI)	Disability *n*, % (95% CI)
Arthritis (*N* = 7,092)	1,279, 18.0% (17.1–19.0)	1,217, 95.1% (93.8–96.2)
Stroke (*N* = 7,087)	153, 2.2% (1.8–2.5)	152, 98.9% (95.4–99.9)
Diabetes (*N* = 7,085)	690, 9.7% (9.6–10.4)	620, 89.9% (87.4–92.0)
Chronic lung diseases (*N* = 7,081)	147, 2.0% (1.7–2.4)	142, 96.4% (92.2–98.9)
Asthma (*N* = 7,085)	343, 4.8% (4.3–5.4)	330, 96.2% (93.6–98.0)
Depression (*N* = 7,086)	131, 1.8% (1.5–2.2)	127, 96.5% (92.4–99.2)
Hypertension (*N* = 6,670)	2,180, 32.7% (31.5–33.8)	1937, 88.9% (87.5–90.2)
Cataract (*N* = 7,081)	1,506, 21.3% (20.3–22.2)	1,389, 92.2% (90.8–93.5)
Edentulism (*N* = 7,086)	895, 12.6% (11.9–13.4)	834, 93.2% (91.3–94.8)
Obesity (*N* = 6,494)	1,229, 18.9% (18.0–19.9)	1,130, 91.9% (90.3–93.4)
Multimorbidity (*N* = 7,118)	2,613, 36.7% (35.6–37.8)	2,515, 96.2% (95.4–97.0)

### Association between disability and multimorbidity

The prevalence of disability increased with increase in age and was found to be highest among the individuals aged 80 years and above 97.5% (95% CI: 95.3–98.9). Female respondents (93.0%) as well as those residing in rural areas (90.5%), with no formal education (93.4%) were found to have highest prevalence of disability. Individuals with multimorbidity (93.0%) had greater prevalence of disability as compared to those who did not have multimorbidity.

Univariate ordinal logistic regression showed that age of respondents, gender, residence, education, occupation, partner status, wealth index and multimorbidity along with self-rated health were significantly associated with disability (Table 3).

**Table 3:** Association between disability and various participants’ characteristics.

Variables		Disability present	
		*n*, %* (95% CI)	Crude odds ratio (95% CI)
Age (Years)	50–59	2,454, 84.5% (83.1–85.8)	Reference
	60–69	2,302, 90.0% (88.8–91.1)	1.68 (1.47–1.92)
	70–79	1,216, 95.2% (93.9–96.3)	2.62 (2.22–3.01)
	≥80	355, 97.5% (95.3–98.9)	7.21 (5.29–9.82)
Sex	Male	2,867, 84.7% (83.5–85.9)	Reference
	Female	3,460, 93.0% (92.1–93.8)	1.84 (1.64–2.10)
Residence	Rural	4,596, 90.5% (89.6–91.3)	1.38 (1.16–1.65)
	Urban	1731, 85.5% (83.9–87.0)	Reference
Education	No formal education	3,125, 93.4% (92.5–94.2)	2.13 (1.90–2.39)
	Been to school	3,202, 85.2% (84.0–86.3)	Reference
Occupation	Never worked	1,655, 93.0% (91.7–94.1)	1.35 (1.19–1.53)
	Worked	4,671, 87.7% (86.8–88.6)	Reference
Partner Status	Have partner	4,643, 87.3% (86.3–88.1)	Reference
	No partner	1,682, 94.4% (93.2–95.4)	2.21 (1.93–2.53)
Wealth Index	Most deprived	1,350, 94.2% (92.9–95.3)	2.41 (2.02–2.87)
	2	1,175, 92.0% (90.4–93.4)	1.99 (1.65–2.39)
	3	1,146, 89.3% (87.4–90.8)	1.63 (1.35–1.99)
	4	1,298, 87.3% (85.3–88.8)	1.28 (1.06–1.54)
	Most Affluent	1,358, 83.6% (81.7–85.3)	Reference
Multimorbidity	Present	2,429, 93.0% (91.9–93.9)	1.75 (1.54–1.97)
	Absent	3,898, 86.8% (85.7–87.7)	Reference
Self-rated health	Very good	186, 64.2% (58.5–69.9)	0.15 (0.11–0.20)
	Good	1781, 80.8% (79.1–82.4)	0.37 (0.32–0.43)
	Moderate	3,092, 93.0% (92.1–93.8)	Reference
	Bad	1,156, 98.4% (97.5–99.0)	4.14 (3.48–4.91)
	Very bad	110, 98.7% (93.7–99.8)	11.16 (6.58–18.94)

Table 4 showed the adjusted association between the disability and various socio-demographic characteristics. Age was found to be significantly associated with disability and an increasing trend was seen with increase in disability. The individuals aged ≥80 years had 4.90 times [AOR: 4.90 (95% CI: 3.50–6.82), *p* < 0.001] more chances of having disability as compared to individuals aged 50–59 years. Females had 76% higher [AOR: 1.76 (95% CI: 1.49–2.08), *p* < 0.001] risk of disability as compared with males. Older adult with no formal education had 35% higher [AOR: 1.35 (95% CI: 1.19–1.54), *p* < 0.001] chances of disability compared to the educated. Older adults with multimorbidity had 1.45 times higher [AOR: 1.45 (95% CI: 1.28–1.66), *p* < 0.001] odds of disability compared with those who did not have multimorbidity. Individuals who self-rated their health as very bad had 10.24 [AOR: 10.24 (95% CI: 5.96–17.59)] times higher chances of having disability compared with those who reported their health as moderate.

**Table 4:** Multiple ordinal logistic regression between disability and various participant’s characteristics.

Variables		Disability present	
		Adjusted odds ratio (95% CI)	*p*-value
Age (years)	50–59	Reference	
	60–69	1.50 (1.31–1.73)	<0.001***
	70–79	2.08 (1.75–2.48)	<0.001***
	≥80	4.90 (3.50–6.82)	<0.001***
Sex	Male	Reference	
	Female	1.76 (1.49–2.08)	<0.001***
Residence	Rural	1.12 (0.94–1.35)	0.204
	Urban	Reference	
Education	No formal education	1.35 (1.19–1.54)	<0.001***
	Been to school	Reference	
Occupation	Never worked	0.90 (0.78–1.03)	0.129
	Worked	Reference	
Partner status	Have partner	Reference	
	No partner	1.11 (0.96–1.29)	0.150
Wealth index	Most deprived	1.62 (1.34–1.96)	<0.001***
	2	1.57 (1.27–1.92)	<0.001***
	3	1.38 (1.14–1.67)	<0.001***
	4	1.11 (0.91–1.35)	0.288
	Most affluent	Reference	
Multimorbidity	Present	1.45 (1.28–1.66)	<0.001***
	Absent	Reference	
Self-rated health	Very good	0.19 (0.15–0.26)	<0.001***
	Good	0.42 (0.37–0.49)	<0.001***
	Moderate	Reference	
	Bad	3.47 (2.93–4.10)	<0.001***
	Very bad	10.24 (5.96–17.59)	<0.001***

*p < 0.05, **p < 0.01, ***p < 0.001.

### Healthcare utilization among older adults in India

Table 5 provides the overall healthcare utilization of multimorbid individuals as well as individuals with multimorbidity and disability. 1,447 (63.6%) of the individuals having disability seeked care from private facilities followed by public facilities 567 (24.9%) whereas only 25 (1.12%) of the respondents utilized health facilities provided by charity.

**Table 5:** Healthcare utilization among older adults in India.

Healthcare utilization	Disability absent (*n*, %*)	Disability present (*n*, %*)	Disability + multimorbidity (*n*, %*)
Private	55 (68.70%)	1,447 (63.60%)	697 (67.80%)
Public	14 (18.20%)	567 (24.90%)	235 (22.80%)
Charity	0 (0.00%)	25 (1.12%)	14 (1.30%)
Home visits	5 (6.50%)	84 (3.70%)	33 (3.20%)
Others	5 (6.60%)	152 (6.70%)	50 (4.80%)
Total	79 (100%)	2,275 (100%)	1,028 (100%)

*Column percentage.

For respondents with disability and multimorbidity, we observed almost 697 (67.8%) individuals visited private sector followed by public sector 235 (22.8%). and the least healthcare utilization was from charitable clinics 14 (1.3%).

## Discussion

Our findings suggest that the prevalence of disability was considerably high among the older adults in India. The individuals aged ≥80 years, older adult with no formal education, those with multimorbidity, and those who self-rated their health as very bad were found to be the significant predictors of disability in India. Hypertension was found to be the most prevalent chronic conditions followed by cataract (21.3%). Current study suggests that for both disability and multimorbidity, the healthcare utilization was highest from private sector followed by public facilities and the least contribution was of charitable clinics.

The overall prevalence of disability was found to be 89.0% (95% CI: 88.3–89.8) in the present study. The result of our study is in line with the findings of study in Punjab which reported the prevalence of disability to be around 87.5% (26). Among the studies that employed the WHODAS 2.0, the occurrence of disability differed depending on the chosen threshold values or cut-off scores for the summary scores. Similar to our study a cross-sectional study conducted in Pune among adults aged 60 years and older employed the WHODAS 2.0 (27). In this study, a summary score above 4 on the WHODAS 2.0 was considered as indicative of disability, with a reported prevalence of 70.4% (27). In the research conducted among older adult individuals aged 75 years and older by Virués-Ortega et al. (28), disability was classified into four levels: no disability (0–4), mild disability (5–24), moderate disability (25–49), and severe/extreme disability (50–100). The age-adjusted standardized prevalence of disability in this population was found to be 39.17% for mild disability, 15.31% for moderate disability, and 10.14% for severe/extreme disability (28). Similar patterns of results were obtained from the study conducted in Spain using WHODAS 2.0 scoring having a prevalence of 49.8% and mild disability being the highest and very severe being the lowest form of disability which are contrary to our findings (29). Similarly, a study conducted in Spain among individuals 50 years and older revealed a prevalence of 51.5, 28.9, and 16.1% for mild, moderate, and extreme/severe disability, respectively, (30). Another study in an urban resettlement area of Delhi reported prevalence figures for mild, moderate, severe, and extreme disability as 28.0, 49, 19.2, and 3.8%, respectively, which is in accordance with the results of this study (31). Comparisons across countries using WHODAS 2.0 tools revealed varying prevalence estimates, a study conducted in Poland reported the extreme disability of around 6.3% which is very high comparable with the present study (32). The present study shows that when comparison with other countries the prevalence of extreme disabilities in low as in India. The complexity and evolving nature of disability results with a variety of definitions and measurement scales being utilized in different studies. This can lead to variations in the reported prevalence of disability. The prevalence of our study is higher than other studies which could be because of the threshold values that we have utilized for this study. However, utilizing the WHODAS 2.0 measurement scale allows for more accurate comparisons across different populations.

With increasing age, the prevalence of disability increases proportionally with individuals aged ≥80 years having the highest prevalence of disability. The results of this study were in concurrence with results of study where high disability burden was seen in individuals aged 70 years and above (27–29). As individuals age, they become more prone to disability due to various factors such as degenerative health conditions, chronic illnesses, falls, and injuries which contribute to the increased susceptibility. Additionally, older adults are at a higher risk for developing substance use problems, neurological and intellectual disorders, and physical impairments such as hearing loss and osteoarthritis (33). As result of aging process, they also experience psychological issues such as a reduced sense of proprioception, difficulty adapting to changes in the environment and social roles, and increased risk of adverse life events (34). All this may lead to compromised quality of life and hence, require greater attention.

Furthermore, we observed the gender differentials for disability with females having higher rate of disability as compared to males. This aligns with the findings of a study where females had a higher rate of disability (31). According to a study, there is a significant difference in health between males and females with the former having better overall health (35). One of the reasons for this could be that women tend to live longer than men, and therefore, there is a higher probability of disability among older women. Additionally, women are more susceptible to certain chronic health conditions such as osteoporosis and rheumatoid arthritis, which makes them more prone for fractures thereby increasing the risk of disability (36, 37). Menopause may be linked to an increased risk of certain health issues such as osteoporosis, cardiovascular disease, and cognitive decline including memory and loss of attention thus indirectly acting as a risk factor for disability (38–40). Older women have more mobility limitations and face more difficulty in performing activities of daily living, such as bathing and dressing, in comparison to older men (41, 42). Furthermore, women are more likely to face social and economic disadvantages, such as poverty and lack of access to healthcare, which can increase the risk of disability (43). Therefore, women’s health especially those having disability needs to be prioritized. Existing programs for women should not be confined to reproductive years instead should target for overall well-being beyond the reproductive health also.

Our research found that individuals with fewer years of schooling had a higher rate of disability. This was consistent with previous studies which also found a direct association between lower levels of education and higher rates of disability (27, 29). Among the community-dwelling older population in China, a study revealed that individuals with fewer years of schooling had high prevalence which is consistent with the findings of this study (44). Despite government policies that provide a quota of 5% for individuals with disabilities in government-aided institutions and 4% in government jobs, the prevalence of disability remains considerably higher among individuals with lower educational levels, highlighting persistent disparities (45). Possible reasons for this may include barriers related to attitudes, lack of inclusivity, difficulties in transportation, and a lack of understanding among parents and caregivers about the importance of education for individuals with disabilities (46).

Furthermore, previous studies have demonstrated that individuals in the lowest wealth strata have a significantly heightened risk of death and disability at all ages compared to those in the highest wealth bracket (47, 48). This aligns with the findings of our current study. A study conducted by Paul et al. (49) suggests that older adults from lower socio-economic backgrounds were more likely to experience difficulties with activities of daily living, instrumental activities of daily living, and functional limitations, indicating a higher prevalence rate and lower recovery rate due to limited access to healthcare resources. Wealth may serve as a predictor for scarce financial resources, which can be exacerbated by factors such as job loss, retirement, or aging (50). Individuals living in poverty may be exposed to hazardous working conditions, which can have a detrimental impact on their health, including disability. Additionally, limited access to healthcare and education may increase their risk of developing disabilities (9, 51). Lesser financial support will also mean reduced healthcare access and compromised quality of life.

Multimorbidity is significantly associated with disability among older adults. The presence of multiple chronic conditions can adversely affect the daily lives of older individuals, leading to higher rates of disability, frailty, and increased healthcare expenses (52). The impact of multiple chronic conditions on quality of life can be greater than the sum of the individual effects of each condition, suggesting potential negative synergistic interactions among the conditions in older adults with multimorbidity (53). Studies using longitudinal data and prospective determination of disability have provided stronger evidence for this association. For instance, study utilizing a count measure of multimorbidity found that a greater number of chronic conditions predicted greater loss of mobility over 18–20-year follow-up period. It was found that changes in mobility were not due to any single condition but rather to the overall burden of multimorbidity (54). The growing body of research highlights the significant role of inflammation in the aging process, suggesting it as a common pathway through which various factors lead to disability and multimorbidity (55). Inflammation is a multifaceted biological response that can be initiated by an array of factors, such as chronic illnesses, lifestyle behaviors, and environmental influences, all of which are prevalent among aging populations (56–58). Furthermore, evidence suggests that inflammation serves as a critical mediator in the interplay between multimorbidity and functional limitations in both middle-aged and older adults (54, 59). This mediation implies that elevated systemic inflammation levels may intensify the physical and cognitive declines associated with multiple chronic conditions, thereby heightening the risk of disability (60, 61). Studies also indicates that the chronic inflammatory state often observed in individuals with multimorbidity not only affects overall health outcomes but also accelerates the decline in functional abilities over time (62). These insights underscore the crucial role inflammation may play in connecting multimorbidity to disability, emphasizing the necessity of targeting inflammatory pathways in interventions designed to enhance health outcomes for older adults (54). Nonetheless, these factors may lead to a bi-directional association between multimorbidity and disability which should not be overlooked (53, 63).

Additionally poor self-rated health is also associated with disability. Poor self-rated health is a strong predictor of disability, and there are several reasons for it. The probable reasons could be that individuals with poor self-rated health may have more chronic health conditions, limited access to healthcare, lower level of physical activity and may have poor nutrition. Additionally, lower level of social support can affect their mental health and lead to disability. Self-rated health is a proxy indicator of quality of life (QoL) and it reflects the need to put efforts to improve the QoL among this group.

We also examined healthcare utilization among individuals with both disability and multimorbidity, as well as those with only disability. The health care utilization was highest in the private sector. The findings are consistent with a study that revealed greater use of private hospitals, as they may have greater access to specialized care and services that are not available in public hospitals (54). Private hospitals may provide specialized services and facilities, such as specialized units for individuals with disabilities, which can offer tailored care and support. This can be particularly useful for older adults with mobility limitations. They also may have more comfortable and accessible facilities. Furthermore, private hospitals may have access to more advanced equipment and technology which can enhance the quality and effectiveness of care for individuals with disabilities or chronic conditions (64, 65). These findings highlight the need to strengthen the primary care so that more and more individuals can access affordable care at doorstep. Moreover, we also observed that individuals with disability were more likely to visit charitable facilities or public hospitals as compared to non-disabled groups. A major reason for this could be that public hospitals are in vicinity and hence easily accessible. Additionally, primary care remains first and foremost point of contact for majority of population in India. Affordability may also be a major factor for higher number of visits at both public and charitable facilities.

### Implications for policy and practice

Our study highlights the critical role of multimorbidity in driving disability among older adults. Addressing this issue requires a coordinated and comprehensive approach to healthcare policy. Policymakers should focus on enhancing healthcare services that are specifically designed to manage multiple chronic conditions concurrently. This includes improving accessibility to healthcare and ensuring that services are well-integrated across various levels of care. Expanding home-based care options, which address the complexities of family level multimorbidity, can enhance the continuity of care and reduce the burden on institutional healthcare settings (66).

Additionally, it is vital to develop public health interventions that focus on prevention and early detection of chronic diseases, particularly among high-risk populations. Strategies such as Information, Education, and Communication (IEC) and Behavior Change Communication (BCC) are essential in promoting healthy behaviors and controlling risk factors like tobacco use, which is a significant contributor to chronic conditions and, subsequently, disability (67). These public health campaigns should be tailored to raise awareness about the impact of multimorbidity on disability and encourage preventive health measures among aging populations.

Further, structural improvements within healthcare systems are needed to address accessibility issues faced by individuals with disabilities. This could include policy efforts to make healthcare facilities more inclusive and accessible to older adults dealing with multimorbidity and disability, ensuring they receive timely and appropriate care. Through these targeted approaches strengthening integrated care, promoting home-based services, enhancing accessibility, and implementing effective public health campaigns—health systems can better address the needs of older adults facing the dual burden of multimorbidity and disability.

### Strength and limitations

The current study possesses both strengths and limitations, which we have thoroughly deliberated upon. The strengths of this study are firstly, it is the first of its kind in India to estimate the prevalence of disability using WHODAS 2.0 scale (a validated and reliable scale, with strong psychometric properties) and to assess its association with multimorbidity among older adults using a nationally representative dataset. Secondly, the scale that is used in the current study covers a wide range of domains of functioning such as physical, cognitive, emotional, and social aspects, offers a broad assessment scale. Aligned with the International Classification of Functioning, Disability and Health (ICF) framework, it also enables a holistic overview of an individual’s functional status. Thirdly, this study also provides an insight into the general prevalence of disability in India, serving as a springboard for more in-depth investigations on the relationship between disability and the development of multimorbidity in the future. Furthermore, it also provides a current estimate of disability among individuals with multimorbidity, serving as a foundation for policy considerations.

However, despite several strengths this study has certain limitations also the scale that is used is based on self-reported data, which may be influenced by an individual’s bias or difficulty in accurately reporting their functional status. Additionally, it may not capture all aspects of disability, particularly those that are not related to physical or mental functioning. The prevalence of various chronic conditions was self-reported by the respondents, which might induce recall bias in the study. When drawing conclusions based on the findings of a cross-sectional study, one should also take into consideration the issue of temporal ambiguity known as “Protopathic bias.”

## Conclusion

This study highlighted that out of every 10 individuals almost nine had some kind of disability among older adults in India. The likelihood of disability escalated with age and was higher among women, and those with no formal education. Older adult individuals were more susceptible to multimorbidity which may lead to disability as these factors are bi-directional. Therefore, policies aimed at improving the well-being of older adults should focus on managing home-based care for multimorbidity and disability. As part of the National Programme for Health Care of the Older adult, the establishment of rehabilitation units at Community Health Centres is envisaged. Nonetheless, it is imperative to ensure the provision of comprehensive health care services at the community level to effectively address geriatric disability. Longitudinal studies using the ICF bio-psycho-social model of disability will be helpful to better address the growing needs of disability.

## Data Availability

The original contributions presented in the study are included in the article/Supplementary material, further inquiries can be directed to the corresponding authors.
