# Supplementary material for: Understanding the association of disability with multimorbidity, and healthcare utilization in India’s older adult population: insights from cross-sectional evidence of SAGE-2
Source: Front Public Health. 2024 Oct 23;12:1435315. doi: 10.3389/fpubh.2024.1435315 (PMC11537888; doi:10.3389/fpubh.2024.1435315)
Supplement: Supplementary file 1 [file Table_1.docx]

**Supplementary files:**

| **Supplementary table 1:** **Description of variables in the study, SAGE wave-2, (2015-16)** | | |
| --- | --- | --- |
| **Outcome variables** | **Question** | **Categories** |
| **Cognition** | Overall, in the last 30 days, how much difficulty did you have in learning a new task? | Recoded into:  1. None  2. Mild  3. Moderate  4.Severe  5. Extreme |
| **Interpersonal relation** | 1. “Overall, in the last 30 days, how much difficulty did you have with making new friendships or maintaining current friendships?”  2. “Overall, in the last 30 days, how much difficulty did you have with dealing with strangers” | Recoded into:  1. None  2. Mild  3. Moderate  4.Severe  5. Extreme |
| **Functional assessment** | 1.“In the last 30 days, how much difficulty did you have in standing for long periods?”  2. “In the last 30 days, how much difficulty did you have in taking care of your household responsibilities?”  3.“In the last 30 days, how much difficulty did you have in joining in community activities?”  4. “In the last 30 days, how much difficulty did you have concentrating on doing something for 10 minutes?”  5.“In the last 30 days, how much difficulty did you have in walking a long distance such as a kilometer?”  6.“In the last 30 days, how much difficulty did you have in bathing/washing your whole body?”  7. “In the last 30 days, how much difficulty did you have in getting dressed?”  8. “In the last 30 days, how much difficulty did you have in your day-to-day work?”  9. “In the last 30 days, how much have you been emotionally affected by your health conditions?”. | Recoded into:  1. None  2. Mild  3. Moderate  4.Severe  5. Extreme |
| **Independent variables** | **Question** | **Categories** |
| **Age (in years)** | How old are you now? | Recoded into:  1.50-59 years  2. 60-69 years  3. 70-79 years  4. ≥80 |
| **Education** | Have you ever been to school? | Recoded into:  1.Yes  2.No |
| **Occupation** | Have you ever in your life done any of these things or any type of work (not including housework)? | Recoded into:  1.Worked  2.Never worked |
| **Partner status** | what is your current marital status? | Recoded into:  1. No partner (never married+separated+divorced)  2.Have partner (Currently married) |
| **MPCE quintiles** | Based on monthly per capita expenditure (MPCE) | Recoded into:  1.Most deprived  2.2  3.3  4.4  5.Most affluent |
| **Alcohol consumption** | have you ever consumed a drink that contains alcohol? | Recoded into:  1.Yes  2.No |
| **Tobacco consumption** | Have you ever smoked tobacco or used  smokeless tobacco? | Recoded into:  1.Yes  2.No |
| **Self-rated health** | In general, how would you rate your health today? | Recoded into:  1.Very good  2.Good  3.Moderate  4.Bad  5.Very bad |
| **Healthcare utilization** | What was the last (most recent) health care facility you visited in the last 12 months? | Recoded into:  1.Private  2.Public  3.Charity  4.Home Visits  5.Others |
